# Supplementary material for: Hierarchical organization of a Sardinian sand dune plant community
Source: PeerJ. 2016 Jul 12;4:e2199. doi: 10.7717/peerj.2199 (PMC4950538; doi:10.7717/peerj.2199)
Supplement: Supplemental Information 2 — Results of three-way ANOVAs considering the species, the substrate (under adult vs bare sand) and the seeds category (conspecific vs other seeds) all as orthogonal and fix factors (Fig. 5). [file peerj-04-2199-s002.docx]

*Appendix*

Table S1. Seeds distribution experiment. Results of three-way ANOVAs considering the species, the substrate (under adult vs bare sand) and the seeds category (conspecific vs other seeds) all as orthogonal and fix factors (Fig. 5).

|  | **Front** | | | **Fore Top** | | | **Middle** | | | **Back** | | |
| --- | --- | --- | --- | --- | --- | --- | --- | --- | --- | --- | --- | --- |
| **Source** | df | F | p | df | F | p | df | F | p | df | F | p |
| Species (sp) | 1 | 0.00 | 0.9965 | 2 | 0.70 | 0.4998 | 1 | 0.35 | 0.5572 | 1 | 0.76 | 0.3861 |
| Substrate (su) | 1 | 6.67 | 0.0118 | 1 | 8.89 | 0.0036 | 1 | 13.27 | 0.0005 | 1 | 8.49 | 0.0048 |
| Seeds category (se) | 1 | 8.36 | 0.0051 | 1 | 0.60 | 0.4395 | 1 | 0.20 | 0.6550 | 1 | 0.61 | 0.4389 |
| spXsu | 1 | 0.10 | 0.7515 | 2 | 0.53 | 0.5905 | 1 | 0.35 | 0.5546 | 1 | 0.75 | 0.3907 |
| spXse | 1 | 0.00 | 0.9439 | 2 | 4.23 | 0.0170 | 1 | 15.74 | 0.0002 | 1 | 10.04 | 0.0022 |
| suXse | 1 | 6.56 | 0.0125 | 1 | 0.22 | 0.6432 | 1 | 0.25 | 0.6188 | 1 | 0.70 | 0.4048 |
| spXsuXse | 1 | 0.11 | 0.7415 | 2 | 3.45 | 0.0354 | 1 | 12.59 | 0.0007 | 1 | 8.34 | 0.0051 |
| RES | 72 |  |  | 108 |  |  | 72 |  |  | 72 |  |  |
