# Supplementary material for: Hierarchical organization of a Sardinian sand dune plant community
Source: PeerJ. 2016 Jul 12;4:e2199. doi: 10.7717/peerj.2199 (PMC4950538; doi:10.7717/peerj.2199)
Supplement: Supplemental Information 3 — Survivorship of natural seedlings marked in October 2013 that survived after 3 seasons in each zone. For each zone and species, the seedlings were marked associated with conspecific adults and in bare substrate > 30 cm from adult vegetation. Carpobrotus seedlings were only found under adult Carpobrotus, so all 50 marked Carpobrotus seedlings were with conspecifics. [file peerj-04-2199-s003.docx]

Table S2. Survivorship of natural seedlings marked in October 2013 that survived after 3 seasons in each zone. For each zone and species, the seedlings were marked associated with conspecific adults and in bare substrate > 30 cm from adult vegetation. *Carpobrotus* seedlings were only found under adult *Carpobrotus*, so all 50 marked *Carpobrotus* seedlings were with conspecifics.

|  |  | ***Lotus*** | | ***Pancratium*** | | ***Armeria*** | | ***Carpobrotus*** | |
| --- | --- | --- | --- | --- | --- | --- | --- | --- | --- |
| Zone | Distance | N° of marked seedlings | Survivorship (%) | N° of marked seedlings | Survivorship (%) | N° of marked seedlings | Survivorship (%) | N° of marked seedlings | Survivorship (%) |
|  |  |  |  |  |  |  |  |  |  |
| Fore | Adjacent bare sand | 25 | 0 | 25 | 0 |  |  |  |  |
|  | Next to adult | 25 | 0 | 25 | 4 |  |  |  |  |
| Middle | Adjacent bare sand | 25 | 4 | 25 | 0 | 25 | 32 |  |  |
|  | Next to adult | 25 | 4 | 25 | 0 | 25 | 12 |  |  |
| Back | Adjacent bare sand |  |  |  |  | 25 | 84 |  |  |
|  | Next to adult |  |  |  |  | 25 | 80 | 50 | 80 |
