# Supplementary material for: Hierarchical organization of a Sardinian sand dune plant community
Source: PeerJ. 2016 Jul 12;4:e2199. doi: 10.7717/peerj.2199 (PMC4950538; doi:10.7717/peerj.2199)
Supplement: Supplemental Information 4 — Two-way ANOVA results with zone and treatment as orthogonal and fix factors (Fig. 7). [file peerj-04-2199-s004.docx]

Table S3. Physical stress alleviation experiment. Two-way ANOVA results with zone and treatment as orthogonal and fix factors (Fig. 7).

| **Source** | **df** | **F** | **p** |
| --- | --- | --- | --- |
| Zone (zo) | 2 | 18.30 | 0.0000 |
| Treatment (tr) | 9 | 1.70 | 0.0917 |
| zoXtr | 18 | 0.87 | 0.6151 |
| RES | 210 |  |  |
