# Supplementary figures and images for: Hierarchical organization of a Sardinian sand dune plant community
Source: PeerJ. 2016 Jul 12;4:e2199. doi: 10.7717/peerj.2199 (PMC4950538; doi:10.7717/peerj.2199)

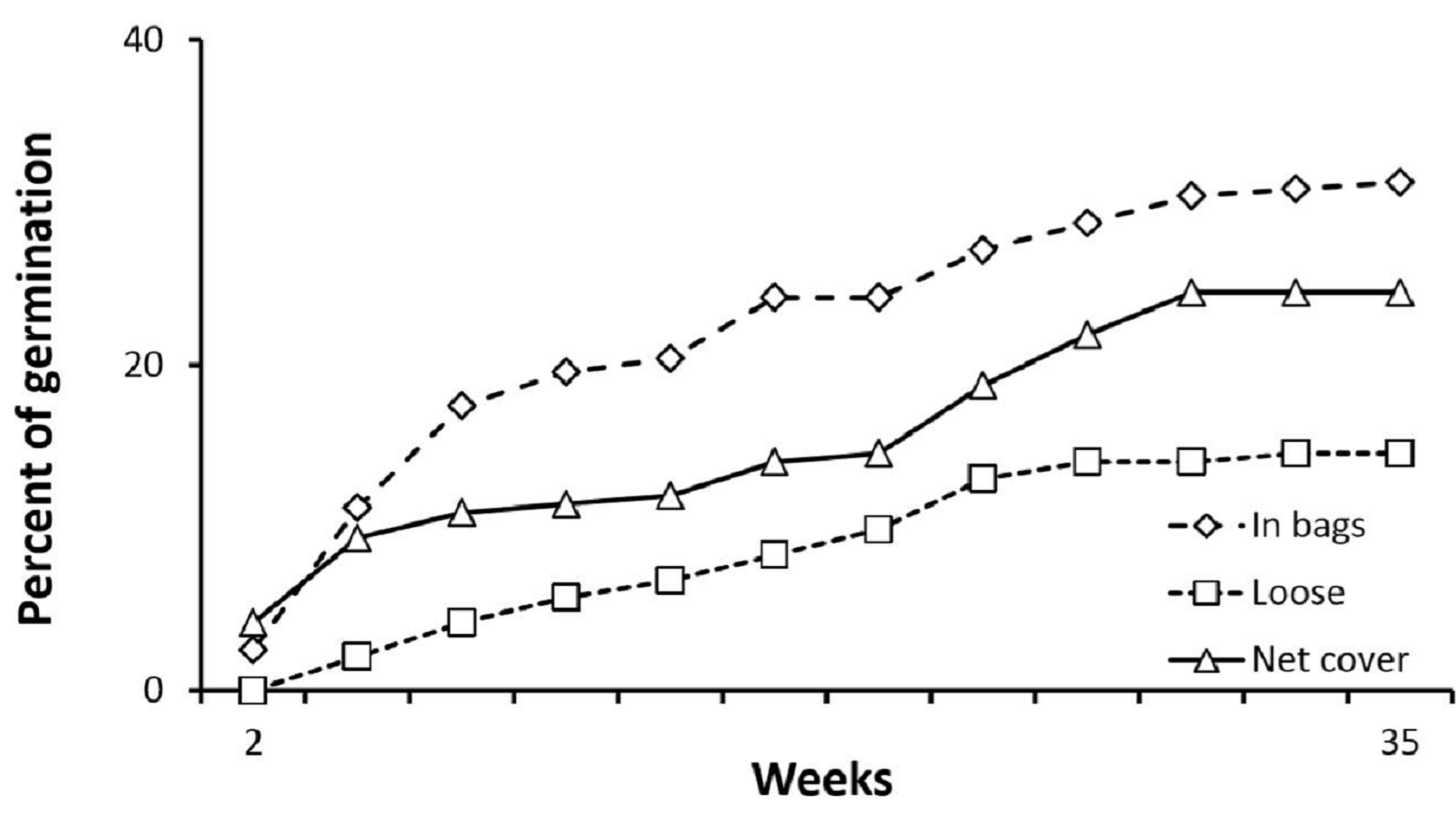

Supplement: Supplemental Information 5 — Germination in the fore-dune where Pancratium seeds were transplanted in germination permeable net bags, loose in the soil and loose in the soil but covered at the surface with nylon mesh to limit erosion (n = 8 treatment). Data are percent germination of the total potential. [file peerj-04-2199-s005.png]
